# Undercarboxylated osteocalcin is associated with vascular function in female older adults but does not influence vascular function in male rabbit carotid artery *ex vivo*

**DOI:** 10.1371/journal.pone.0242774

**Published:** 2020-11-25

**Authors:** Alexander Tacey, Cassandra Smith, Mary N. Woessner, Paul Chubb, Christopher Neil, Gustavo Duque, Alan Hayes, Anthony Zulli, Itamar Levinger

**Affiliations:** 1 Institute for Health and Sport (IHES), Victoria University, Melbourne, VIC, Australia; 2 Australian Institute for Musculoskeletal Science (AIMSS), The University of Melbourne and Western Health, St Albans, VIC, Australia; 3 PathWest Laboratory Medicine WA, Fiona Stanley Hospital, Murdoch, WA, Australia; 4 Medical School, University of Western Australia, Perth, WA, Australia; 5 Department of Medicine-Western Health, Melbourne Medical School, The University of Melbourne, Melbourne, VIC, Australia; 6 Department of Cardiology, Western Health, Melbourne, VIC, Australia; Max Delbruck Centrum fur Molekulare Medizin Berlin Buch, GERMANY

## Abstract

**Background:**

There are conflicting reports on the association of undercarboxylated osteocalcin (ucOC) in cardiovascular disease development, including endothelial function and hypertension. We tested whether ucOC is related to blood pressure and endothelial function in older adults, and if ucOC directly affects endothelial-mediated vasodilation in the carotid artery of rabbits.

**Methods:**

In older adults, ucOC, blood pressure, pulse wave velocity (PWV) and brachial artery flow-mediated dilation (BAFMD) were measured (n = 38, 26 post-menopausal women and 12 men, mean age 73 ± 0.96). The vasoactivity of the carotid artery was assessed in male New Zealand White rabbits following a four-week normal or atherogenic diet using perfusion myography. An ucOC dose response curve (0.3–45 ng/ml) was generated following incubation of the arteries for 2-hours in either normal or high glucose conditions.

**Results:**

ucOC levels were higher in normotensive older adults compared to those with stage 2 hypertension (p < 0.05), particularly in women (p < 0.01). In all participants, higher ucOC was associated with lower PWV (p < 0.05), but not BAFMD (p > 0.05). In rabbits, ucOC at any dose did not alter vasoactivity of the carotid artery, either following a normal or an atherogenic diet (p > 0.05).

**Conclusion:**

Increased ucOC is associated with lower blood pressure and increased arterial stiffness, particularly in post-menopausal women. However, ucOC administration has no direct short-term effect on endothelial function in rabbit arteries. Future studies should explore whether treatment with ucOC, *in vivo*, has direct or indirect effects on blood vessel function.

## Introduction

The bone derived hormone osteocalcin (OC) is a vitamin K-dependent protein that exists in several biological forms. The post-translational γ-carboxylation of less than three glutamic acid residues produces undercarboxylated osteocalcin (ucOC), which has a low affinity for hydroxyapatite and is predominantly found in circulating blood [1]. In recent years ucOC has been suggested as a mediator of a cross-talk between bone and metabolic outcomes [2]. In humans, higher levels of ucOC are associated with a reduced risk of metabolic syndrome and type II diabetes [3–5]. Similarly, ucOC has been reported to improve glucose regulation, adiposity and insulin sensitivity in animal models [6–8]. However, not all studies are in agreement [9, 10]. Given these findings, it is of interest to investigate whether ucOC is involved in other biological functions within the body [11, 12]. As metabolic and cardiovascular diseases (CVD) share common pathological links [13], it is of particular interest to examine the interaction of ucOC with endothelial function and atherosclerosis progression. This is important, not only in the context of CVD, but also because ucOC could be targeted as a future therapy for metabolic diseases.

The association between OC and its isoforms with CVD in humans remains unknown [14, 15]. A number of cross-sectional studies have reported that higher circulating total OC (tOC) is associated with improved vascular health and function [16–18]. Yet, others have reported that higher tOC has adverse [19–21], or even no association [22], with vascular health. However, only a limited number of studies have investigated the role of ucOC in the vasculature. As ucOC is suggested to be the active circulating form of OC, it is particularly important to investigate whether ucOC is associated with vascular function, and if so, whether these effects are beneficial or detrimental.

In animal models, administration of tOC and ucOC *in vivo* improve blood vessel function. For example, daily tOC (30ng/gram) injections for 12 weeks significantly improved pulse wave velocity (PWV), a measure of arterial stiffness, in rats with induced diabetes mellitus [23]. Daily tOC also enhanced vasodilation *ex vivo* in the aorta of apolipoprotein E^-/-^ mice [24]. In another study, 30ng/gram of ucOC administered for 10 weeks in female C57BL/6 mice produced an increase in nitric oxide availability, a key vasoactive molecule [25]. While these *in vivo* studies indicate potential links between OC administration and improvements in vascular health, they also reported concurrent improvements in metabolic outcomes, such as improved glycaemic control and lower adiposity. Therefore, it is unclear whether the improvement in blood vessel function resulted from a direct effect of OC, or an indirect effect from improved metabolic outcomes.

The aims of this study were to a) investigate the association of circulating ucOC levels with endothelial function, arterial stiffness and blood pressure (BP) in older adults via a cross-sectional analysis, and b) examine the direct effect of ucOC on endothelial function in rabbit arteries.

## Methods

### Human participants

Twenty six healthy, community dwelling post-menopausal women (mean age of 73 years) and 12 older men (mean age of 74 years) participated in this study. Inclusion criteria included adults over 60 years old and women >12 months post-menopause. Exclusion criteria included a current diagnosis of diabetes, a body mass index (BMI) over 40kg/m^2^, a fracture within the last 3 months or participation in resistance exercise >2 days per week. Participants were on a range of medications to control for hypertension, cholesterol and CVD, however all were stable and controlled for at least 3 months as per their medical records. Each participant received written and verbal explanations about the nature of the study before signing an informed consent document. This study was approved by Melbourne Health and Victoria University Human Research Ethics Committees. The data were collected as part of a larger clinical trial (ACTRN12618001756213).

### Blood pressure (BP) and vascular function

Brachial artery systolic BP, diastolic BP and mean arterial pressure measurements were recorded using the non-invasive SphygomoCor-XCEL® (AtCor Medical, Sydney, NSW, Australia) diagnostic system. Two measurements were captured, with the lower of the two readings recorded. If the two BP readings were >6 mmHg apart, a third measure was recorded to ensure a true resting value and the average of the two lowest BP measurements were recorded. Participants were split into groups based on hypertension guidelines; normal (<130mmHg/<80mmHg) n = 7, stage 1 hypertension (130–139mmHg/80-89mmHg) n = 14 or stage 2 hypertension (>140mmHG/>90mmHG) n = 17 [26]. None of the participants with normal BP were taking antihypertensive medication, five of the participants with stage 1 hypertension and 10 of the participants with stage 2 hypertension were taking antihypertensive medication. Arterial stiffness was measured by PWV using the subtraction method, with the thigh cuff placed on the thigh and a tonometer used to measure the carotid artery waveform (SphygomoCor-XCEL®) [27].

Endothelial function was assessed via brachial artery flow mediated dilation (BAFMD) using a high-resolution ultrasound (Terason, LifeHealthcare, New South Wales, Australia) with R wave trigger. Brachial artery diameter was assessed for ~10 seconds at baseline (in duplicate and averaged) and during forearm occlusion. Brachial artery diameter was continuously captured after the occlusion cuff release for ~2 minutes (reactive hyperaemia). Peak change was calculated as the peak percentage change in brachial artery diameter from baseline to immediately following peak hyperaemia [28].

### Circulating osteocalcin measurements

Serum samples were taken in the morning following an overnight fast and stored at -80°C until analysis. Serum tOC was measured using an automated immunoassay (Elecsys 170; Roche Diagnostics) [29]. Serum ucOC was measured using the hydroxyapatite binding method, a commonly used, well established method [30]. Each sample was measured once and the inter-assay coefficients of variation were 5.4% and 9.2% for tOC and ucOC, respectively.

### Animals

Male New Zealand White rabbits at 12 weeks of age were randomised into either a normal chow diet (n = 7) (Guinea pig and rabbit pellets, Specialty Feeds, Australia) or an atherogenic diet (n = 10) (a normal diet combined with 1% methionine, 0.5% cholesterol and 5% peanut oil (#SF00-218, Specialty Feeds, Australia)) for 4 weeks [31]. This atherogenic diet has previously been reported to cause endothelial dysfunction in rabbits [31, 32]. The rabbits were housed in individual cages on a 12-hour light/dark cycle at 21°C, with access to water and their assigned chow diet *ad libitum*. At the completion of the 4-week diet, the rabbits were sedated (0.25mg/kg medetomidine) and anaesthetised (4% isoflurane) before exsanguination via severing of the inferior vena cava. The arterial system was immediately flushed with ice cold Krebs solution ((mM) 118 NaCl; 4.7 KCl; 1.2 MgSO_4·_7H_2_O; 1.2 KH_2_PO_4_; 25 NaHCO_3_; 1.25 CaCl and 11.7 glucose) and the carotid arteries were carefully dissected and placed in Krebs solution. The animal experiments were approved by the Victoria University Animal Ethics Committee (#14/005) and complied with the Australian National Health and Medical Research Council code for the care and use of animals for scientific purposes (8^th^ edition).

### Perfusion myography

The carotid arteries were cleaned of connective tissue and fat, with care taken to avoid damaging the arterial wall and the endothelium. Arterial branches were identified, and the carotid arteries were cut to a length of 15–20mm, ensuring no branches were present. The arteries were placed in individual chambers within a perfusion myography system (Zultek Engineering, Melbourne, Australia). Each artery was immersed in either normal Krebs solution (11mM glucose) or a high glucose Krebs solution (20mM glucose) as previously described [32]. The organ baths were warmed to 37°C and bubbled with 95% oxygen and 5% carbon dioxide and were refreshed every 30 minutes over a 2-hour period with the respective Krebs solution. Subsequently, the arteries were cannulated, and the respective Krebs solution pumped through the artery while pressure transducers monitored the intraluminal pressure of the vessel.

The carotid arteries were constricted with phenylephrine (3x10^-7^M), which was added intraluminally and extraluminally. Once a stable constriction was achieved, a dose response curve was completed to ucOC (0.3, 3, 30 and 45ng/ml) (Glu13, 17, 20, osteocalcin (1–46) (mouse) trifluoroacetate salt (Auspep, Australia, H-6552.0500)) or to Krebs (control), each concentration was administered internally via the endothelium and separated by 2 minute intervals. The same mouse ucOC has previously been shown to improve relaxation in rabbit arteries [33]. Following the dose response curve a bolus of acetylcholine (ACh) (10^-5^M) was added internally and two minutes later a bolus of sodium nitroprusside (SNP) (10^-5^M) externally, to determine the maximal endothelium-dependent and endothelium-independent relaxation, respectively. The vasoactive response of the vessels were analysed using the MEDIDAQ software program (MEDIDAQ, Melbourne, Australia). The vasoactivity of the carotid artery was measured as percentage change from the phenylephrine peak pressure and compared to the baseline pressure. Area under the curve (AUC) was calculated as the total relaxation below the phenylephrine plateau caused by the dose response curve, ACh and SNP bolus doses. The endothelium-dependent E_max_ was considered as the relaxation produced by ACh, and the endothelium-independent E_max_ was considered as the relaxation produced by SNP.

### Statistical analysis

Human data were analysed using Statistical Package for the Social Sciences (SPSS, Inc. Chicago, IL, USA, version 22). A one-way analysis of variance (ANOVA) was used to examine the difference in ucOC concentration when participants were split into groups based on BP levels. Spearman rho correlations were used to examine the correlation between ucOC and measures of vascular function (BP, BAFMD and PWV) in all participants. Spearman partial correlations were used for the additional adjustments of age, BMI or age and BMI, as they are strong influencers of ucOC levels [1, 29].

Animal data were analysed using Graphpad prism (version 7.1, Graphpad software Inc, USA). A one-way ANOVA was used to examine the effect of the ucOC dose response curves in rabbit carotid artery segments. AUC was calculated as the total area of relaxation below the maximum phenylephrine pressure and a one-way ANOVA was used to determine the difference in AUC between the ucOC dose response curves. All data is reported as mean ± SEM and statistical analysis was conducted at the 95% confidence level of significance (p < 0.05). Trends were reported when p = 0.05–0.099.

## Results

### Human data

**Fig 1:**
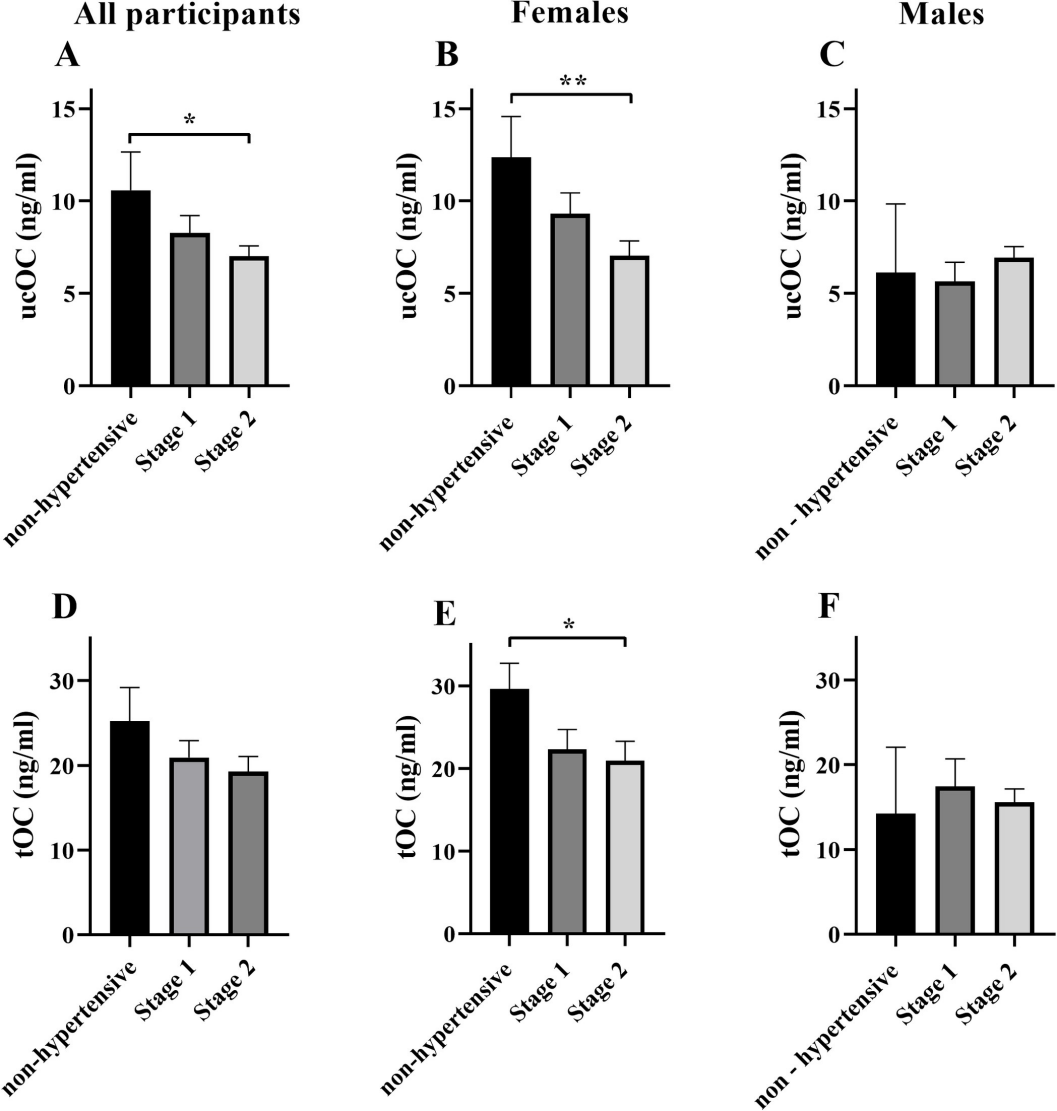
Concentration of ucOC and tOC based on hypertension category. ucOC concentration in all participants (A), women (B) and men (C) and tOC concentration in all participants (D), women (E) and men (F) split into groups based hypertension category; non-hypertensive (<130/<80mm/Hg) (women n = 5, men n = 2), stage 1 hypertension (130-139/80-89mm/Hg) (women n = 10, men n = 4) and stage 2 hypertension (>140/>90mm/Hg) (women n = 11, men n = 6). Given the small sample size in each group, particularly for males, the data are not conclusive and further examination of sex specific effects should be explored. All data mean ± SEM. *p < 0.05, **p < 0.01 between groups. *Abbreviations*: *ucOC; undercarboxylated osteocalcin*, *tOC; total osteocalcin*.

Participant characteristics are presented in Table 1. In older adults with stage 2 hypertension ucOC was reduced by 34% compared to normotensive individuals (p < 0.05, Fig 1A). When split by sex, ucOC was reduced by 43% (p < 0.01, Fig 1B) and tOC was reduced by 30% (p < 0.05, Fig 1E) in women with stage 2 hypertension compared to normotensive women. There was no difference between groups in older men (p > 0.05, Fig 1C and 1F).

**Table 1:** Baseline characteristics.

Variable	n	Mean ± SEM
Participant number (n) [F/M]		38 [26/12]
Age (years)	38	73 ± 0.96
BMI (kg/m^2^)	38	28 ± 0.59
Waist circumference (cm)	36	91 ± 1.58
Currently smoking (n) [%]	38	2 [5]
Caucasian ethnicity (n) [%]	38	38 [100]
Cholesterol medication (n) [%]	38	13 [34]
Antihypertensive medication (n) [%]	38	15 [39]
Heart disease medication (n) [%]	38	10 [26]
tOC (ng/ml)	37	21 ± 1.31
ucOC (ng/ml)	37	8 ± 0.61
ucOC/tOC ratio	37	0.39 ± 0.01
Systolic BP (mmHg)	38	139 ± 2.53
Diastolic BP (mmHg)	38	79 ± 1.43
MAP (mmHg)	38	98 ± 1.74
PWV (m/s)	34	8 ± 0.28
BAFMD–peak dilation (%)	29	4.62 ± 0.44
BAFMD–time to peak dilation (s)	29	58 ± 2.56

Abbreviations: BMI; Body mass index, tOC; total osteocalcin, ucOC; undercarboxylated osteocalcin, BP; blood pressure, MAP; mean arterial pressure, PWV; pulse wave velocity, BAFMD; brachial artery flow mediated dilatation.

### Correlation between ucOC and vascular function outcomes

In the unadjusted model, high circulating ucOC was associated with lower systolic BP and PWV with all participants combined (p < 0.05 for both, Table 2). In women only, higher levels of circulating ucOC and tOC was associated with lower systolic BP (p < 0.01). There were trends for associations between lower MAP and PWV with higher levels of ucOC in women (p = 0.05–0.09 for both, Table 2). When adjusted for age, higher ucOC was associated with lower diastolic BP in all participants and with lower systolic BP in women (p < 0.05 for both, Table 2). Increased ucOC levels tended to correlate with both lower DBP and MAP in women after adjusting for age (p = 0.05–0.09 for both, Table 2). Adjusting for BMI, and BMI and age together, removed all associations of ucOC and tOC with BP and PWV outcomes (p > 0.05). There were no significant correlations between ucOC and tOC with BAFMD peak % dilation in any model, and ucOC or tOC was not associated with any vascular function outcome in men (p > 0.05).

**Table 2:** Correlation of ucOC and tOC with vascular function outcomes.

	ucOC			tOC		
	All (n = 38)	Women (n = 26)	Men (n = 12)	All (n = 38)	Women (n = 26)	Men (n = 12)
SBP						
Model 1	-0.39*	-0.58**	0.25	**-0.31**^	-0.4*	0.18
Model 2	-0.28	-0.48*	0.01	-0.23	**-0.39**^	0.07
Model 3	0.15	-0.07	0.39	0.18	-0.004	0.37
Model 4	0.25	0.05	0.26	0.2	0.04	0.41
DBP						
Model 1	-0.21	-0.3	0.12	-0.23	-0.22	-0.07
Model 2	-0.41*	**-0.44^**	-0.51	-0.34	-0.3	-0.42
Model 3	-0.04	-0.14	-0.05	-0.08	-0.02	-0.4
Model 4	-0.13	-0.14	-0.75	-0.09	-0.02	-0.58
MAP						
Model 1	-0.2	**-0.35^**	0.18	-0.19	-0.24	-0.06
Model 2	-0.32	**-0.44^**	-0.06	-0.25	-0.33	-0.24
Model 3	0.12	-0.05	0.05	0.1	0.03	-0.19
Model 4	0.09	-0.01	-0.42	0.1	0.04	-0.22
PWV						
Model 1	-0.41*	**-0.41^**	-0.32	-0.25	-0.32	-0.01
Model 2	-0.18	-0.26	0.25	-0.14	-0.26	0.4
Model 3	-0.03	0.02	-0.23	0.11	0.003	0.37
Model 4	0.19	0.13	0.54	0.16	0.03	0.76
BAFMD peak %						
Model 1	0.14	0.00	0.39	0.23	0.09	0.29
Model 2	0.22	0.13	0.32	0.26	0.16	0.63
Model 3	0.02	-0.18	0.51	0.13	-0.06	0.46
Model 4	0.05	-1.14	0.29	0.14	-0.04	0.64

Model 1—unadjusted; Model 2—adjusted for age; Model 3—adjusted for BMI; Model 4—adjusted for age and BMI. Given the small sample size in each group, particularly for males, the data are not conclusive and further examination of sex specific effects should be explored.

*p < 0.05

**p < 0.01

**^**p 0.05–0.09 ucOC and tOC vs vascular function outcome.

Abbreviations: ucOC; undercarboxylated osteocalcin, tOC; total osteocalcin SBP; systolic blood pressure, DBP; diastolic blood pressure, PWV; pulse wave velocity, BAFMD; brachial artery pulse wave velocity.

### Perfusion myography

**Fig 2:**
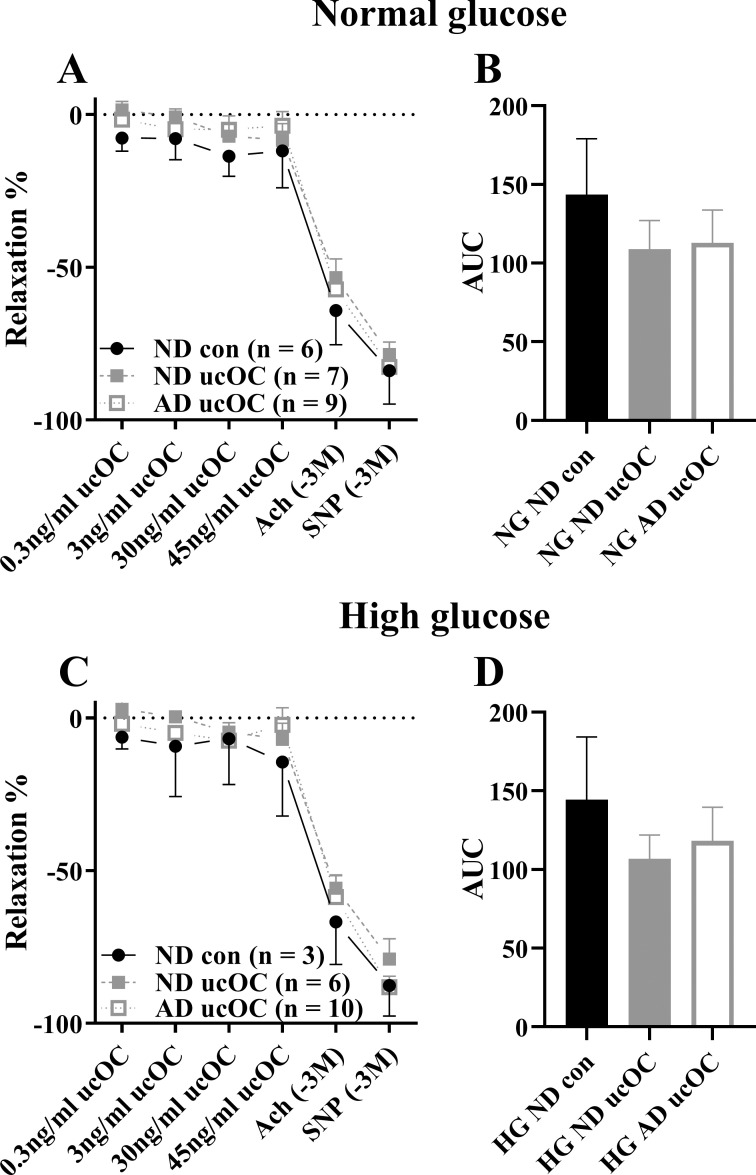
ucOC administration to carotid artery following 2-hour incubation in NG or HG solution. (A) ucOC dose response curve in carotid artery incubated in NG solution and (B) AUC of dose response curve. (C) ucOC dose response curve in carotid artery incubated in HG solution and (D) AUC of dose response curve. All data mean ± SEM. No significant differences were detected. *Abbreviations*: *ucOC*, *undercarboxylated osteocalcin; NG*, *normal glucose media; HG*, *high glucose media; ND*, *normal diet; AD*, *atherogenic diet; Ach*, *acetylcholine; SNP*, *sodium nitroprusside*.

The carotid artery segments from the animals fed the atherogenic diet did not exhibit a reduction in endothelium dependent relaxation in comparison to the arteries from the normal diet fed animals (p > 0.05). The carotid artery vasoactive response from rabbits fed a normal or atherogenic diet and treated *ex vivo* with ucOC was unaltered in both normal and high glucose environments (p > 0.05, Fig 2A and 2C). The endothelium-dependent (ACh) and endothelium-independent (SNP) E_max_ were also unaltered following ucOC treatment, in comparison to the control, suggesting ucOC did not enhance the maximal relaxation of the vessel (p > 0.05, Fig 2A and 2C). The AUC was unaltered by ucOC treatment following both the normal and atherogenic diet and incubation in normal and high glucose conditions (p > 0.05, Fig 2B and 2D).

## Discussion

The major findings of the current study are a) in humans, higher circulating ucOC is associated with lower BP and increased arterial stiffness, which is particularly evident in post-menopausal women, and b) ucOC treatment has no beneficial, but also no adverse, effect on carotid artery function from rabbits fed a normal or atherogenic diet, or exposed acutely to normal and high glucose environments.

A number of studies have examined the correlation of tOC with vascular health and function outcomes. It was reported that tOC was lower in men, but not women with hypertension (aged 24–78 years) [34]. Further, in 3,604 middle to older aged men and women, higher levels of tOC were associated with lower PWV in men, but higher PWV in women. However, when controlled for age and menopause status there was no longer an association between tOC and PWV in women [35]. In middle and older aged men, but not post-menopausal women, higher tOC was associated with lower brachial artery PWV and intima media thickness (IMT), even after adjustment for confounding variables including age and BMI [36]. Yet, not all studies are in agreement, as higher tOC levels were associated with increased IMT, carotid plaque and aortic calcification in middle to older-aged women, but not men [19]. Overall, the findings are conflicting, and this appears to be largely driven by the differences between men and women. A major limitation of these studies is that they do not report the concentration of the individual forms of OC, in particular ucOC, which is important as ucOC is the putative bioactive form of the hormone.

Evidence examining the association of ucOC with vascular function is lacking, but crucial, if we are to understand the role of ucOC in CVD, specifically hypertension and atherosclerosis. In the current study we report that higher levels of ucOC are associated with lower BP in post-menopausal women, but not in older men. However, the relatively small sample size of men in the current study means that definitive conclusions cannot be established. However, similar to the current study, a previous study in older men and women (mean age 64 years old), reported that those with a higher cardiovascular risk score had increased MAP and lower circulating ucOC levels [4]. Conflictingly, in 162 community dwelling men (mean age 48 years old) and women (mean age 55 years old), ucOC was not correlated with systolic BP or diastolic BP [37]. The conflicting outcomes may be related to the age difference between the study cohorts, as age is an important factor in determining ucOC levels [29]. Furthermore, hormone variations between sexes and between pre- and post-menopausal women may also explain some of the diverse findings reported. Overall, whether ucOC is a mediator or a marker of CVD processes requires further investigation. In addition, taking into account several factors including sex, age and hormonal status will be important considerations for future studies.

As the association of ucOC with vascular function in humans is unclear, and given the exact biological functions of ucOC are yet to be fully elucidated, examining its bioactive effect on the vasculature in animal models is important. The most commonly used method of examining the vasoactivity of blood vessels *ex vivo* is via isometric tension analysis. However, in this study, we have utilised a novel perfusion myography system. This technique utilises haemodynamic forces such as shear stress, pressure and pulsatile flow, which are mechanical factors important in the regulation of normal endothelial function, thus creating a more physiological environment [38]. We found that ucOC did not directly influence the vasoactivity of isolated rabbit carotid arteries in either normal or high glucose solutions following an atherogenic or normal diet. A potential limitation of this study is that the atherogenic diet did not cause endothelial dysfunction. This suggests that the carotid artery may be resistant to the development of endothelial dysfunction, as previous studies have reported that the same atherogenic diet caused endothelial dysfunction after 4-weeks in rabbit aorta, iliac and mesenteric arteries [31, 32]. Carotid arteries were used in this study as they lack arterial branches, allowing effective cannulation and perfusion, which would not have been possible in other vessels due to the presence of branches. Notwithstanding, ucOC did not influence vasoactivity when vessels were exposed to a high glucose solution. In support of this, a previous *ex vivo* study, utilising the isometric tension analysis technique, reported similar findings to the current study. The administration of ucOC (10ng/ml and 30ng/ml) to rabbit aorta following an atherogenic diet or normal diet, with incubation in normal or high glucose solution, did not influence endothelium-dependent or endothelium-independent vasodilation [39]. Whilst another study reported that ucOC caused a slight enhancement in ACh-induced endothelium-dependant relaxation in dysfunctional rabbit aorta following an atherogenic diet, this did not occur after a normal diet, suggesting that ucOC may only function to enhance endothelium-dependent vasodilation in a dysfunctional state [33]. However, this requires further investigation. Overall, whilst we report a correlation between ucOC and BP in post-menopausal women, the *ex vivo* data indicates that ucOC has minimal direct biological influence on vascular function. There are several potential reasons for these findings. Firstly, ucOC may not act directly on the vasculature, and the associations observed in some studies may be through indirect pathways, such as via improvements in glycaemic control. Secondly, given recent reports, ucOC may not be as biologically active outside of the skeleton as initially suggested [9, 10].

This study has several limitations. Firstly, the relatively small sample size of older adult men means that definitive conclusions on the association of ucOC with vascular function in males cannot be made. Further research should examine in detail the potential association of ucOC with vascular function in females and males, taking into account confounding variables such as age and BMI. Secondly, a number of human participants were on hypertensive medication which may have influenced their BP measurement, highlighting the important role animal models can play in determining any direct effects of ucOC. Finally, due to only male rabbits being studied, the direct effect of ucOC on endothelial function in arteries from female rabbits is unclear.

In conclusion, increased ucOC is associated with lower BP and arterial stiffness in post-menopausal women, but has no direct effect on endothelial function in rabbit carotid arteries. Future studies should explore whether treatment with ucOC *in vivo* has direct or indirect effects on blood vessel function.
